# Effects of a Ciliate Protozoa Predator on Microbial Communities in Pitcher Plant (*Sarracenia purpurea*) Leaves

**DOI:** 10.1371/journal.pone.0113384

**Published:** 2014-11-25

**Authors:** Taylor K. Paisie, Thomas E. Miller, Olivia U. Mason

**Affiliations:** 1 Department of Earth, Ocean and Atmospheric Science, Florida State University, Tallahassee, Florida, United States of America; 2 Department of Biological Science, Florida State University, Tallahassee, Florida, United States of America; Auburn University, United States of America

## Abstract

The aquatic communities found within the water filled leaves of the pitcher plant, *Sarracenia purpurea*, have a simple trophic structure providing an ideal system to study microscale interactions between protozoan predators and their bacterial prey. In this study, replicate communities were maintained with and without the presence of the bactivorous protozoan, *Colpoda steinii*, to determine the effects of grazing on microbial communities. Changes in microbial (Archaea and Bacteria) community structure were assessed using iTag sequencing of 16S rRNA genes. The microbial communities were similar with and without the protozoan predator, with>1000 species. Of these species, Archaea were negligible, with Bacteria comprising 99.99% of the microbial community. The Proteobacteria and Bacteroidetes were the most dominant phyla. The addition of a protozoan predator did not have a significant effect on microbial evenness nor richness. However, the presence of the protozoan did cause a significant shift in the relative abundances of a number of bacterial species. This suggested that bactivorous protozoan may target specific bacterial species and/or that certain bacterial species have innate mechanisms by which they evade predators. These findings help to elucidate the effect that trophic structure perturbations have on predator prey interactions in microbial systems.

## Introduction

The effect of predators on the prey community is a well-studied area of ecology (e.g., [1]). Predators generally reduce the abundance of prey, which can, in turn, affect the abundance of predators and result in oscillations between predator and prey. Predators can cause prey extinction or prevent prey from colonizing new habitats. Predators can also control biodiversity through either selective feeding by the predators, differential responses of the prey, or indirect interactions through prey species (e.g., keystone predation and trophic cascades).

However, little is known about the effects of predators on microbial communities [2],[3]. Predation by bactivores is difficult to observe *in situ*; therefore, it is not often studied. Further, the rich diversity of microorganisms creates methodological challenges, rendering it difficult to predict overall effects of predation on microbial community structure. It is unknown if standard ecological predictions of predator-prey dynamics and effects of predators on prey community composition will apply in highly speciose microbial communities [4].

There is significant potential for predators to affect microbial community structure. Prior studies have shown that predation by protists can be a dominant factor controlling total bacterial abundances [5],[6]. Bacteria consumed by predators such as protozoans and rotifers are primarily captured through filter feeding and grazing on particles [7]. This predation can be size-selective [5], with possible escape from predation by both smaller [8] and larger size classes of Bacteria. Other bacterial traits, such as motility and shape, may also generate differences in predation of Bacteria by protozoa [5],[8],[9].

Simek et al. [10] used size fractionation and denaturing gradient gel electrophoresis (DGGE) to demonstrate that the protozoa community did affect the bacterial community composition. Using similar methods, Weinbauer et al. [11] also found that protozoa can significantly reduce species diversity (number of DGGE bands). Predators can also affect the relative abundances of Bacteria and, thus, bacterial community composition through selective feeding [6],[12]. For example, prior studies have shown that filamentous Bacteria can evade predation, allowing them to dominate bacterial communities when predators are present [13]. The advent of high-throughput sequencing technologies, such as iTag sequencing of 16S rRNA genes, provides important new tools for more highly resolved characterization of microbial community structure in the context of predator-prey interaction.

To examine microscale predator-prey interactions, we used the microcosms [14] found in water-filled leaves of the carnivorous pitcher plant, *Sarracenia purpurea*. These inquiline communities have a simple trophic structure [15], making them ideal for evaluating the influence protozoa predation has on a natural microbial community. During the growing season, *S. purpurea* makes new leaves ∼ every 4 weeks; prior to opening, the leaves are sterile [16]. The leaves fill with rainwater and produce nectar on the lip, attracting insects, primarily ants, that fall in the water and drown [17]. The water in the leaf is rapidly colonized by a number of other specialist species [18], including a high diversity of Bacteria [19],[20]. Prey that are trapped in the leaves of the pitcher plant are broken down by detritivores, which are consumed by bactivorous protozoans and rotifers (see review in [17]). These bacterivores are consumed by the mosquito larva, *Wyeomyia smithii* [20]. Interactions between the invertebrate and microbial community were previously documented, including “cascading” effects of mosquito larvae on bacterial diversity [16].

The microbial communities in the pitcher plant, *S. purpurea*, have been described in several previous studies. In *S. purpurea* pitcher fluids, Krieger and Kourtev [20] analyzed 16S rRNA genes using DGGE and sequencing, while Gray et al. [19] used cloning and sequencing; both reported that Alphaproteobacteria, Betaproteobacteria and Bacteroidetes were dominant. Peterson et al. [16] used terminal restriction fragment length polymorphism (TRFLP) to analyze the microbial diversity in *S. purpurea*'s pitcher fluids and suggested that the microbial community is influenced by, among other factors, the presence or absence of bacterial predators.

Here we used iTag sequencing of archaeal and bacterial16S rRNA genes from the fluid of pitcher plants maintained with and without the protozoan, *Colpoda steinii*. A total of 1.3 million 16S rRNA gene sequences were generated and analyzed. This method provides a highly resolved platform to examine microscale effects of predators on prey community structure. Further, the high-sequence coverage allows for analysis of a large number of sample replicates. In this paper, we provide a preliminary description of the Archaea and Bacteria found in pitcher plants. Further, we describe the effect of a single protozoan predator on patterns of relative abundance of species within the microbial community.

## Materials and Methods

### Sample Collection and Processing

The microbial samples of pitcher plant fluids were obtained from the Apalachicola National Forest, which is maintained by the National Forest Service, US Department of the Interior. No permissions were required to remove microbial water samples from the pitcher plant leaves and none of the microbial species used in this study are listed as endangered or threatened. Approximately 50 plants were chosen haphazardly from across Crystal Bog (30°11′N, 84°54′W). Fluid (10–15 ml) was extracted asceptically from a randomly chosen single leaf per plant, avoiding leaves that had red or pink tainted fluid, indicating anoxia, and brown and dying leaves to prevent anomalies associated with older leaves [18]. The fluid was immediately cooled and transported to the lab, where it was combined into one flask and sequentially filtered through 200, 50, and 4 µM filters using aseptic techniques. After filtration, the broth was inspected for protozoa or anything other than Bacteria and Archaea by censusing three samples of 0.01 ml using a phase-contrast microscope at 100×.

The study was conducted in greenhouses at Florida State University using plants obtained from the Apalachicola National Forest under Forest Products Free Use Permit No. 5713 to T. E. Miller. Newly opened leaves capable of holding 15–20 ml of fluid were identified on separate plants. Aliquots of the filtered microbial broth (10 mL) were then placed in 20 of these newly opened leaves, along with two dead ants (*Solonopsis invicta*), a common prey found in pitcher plants in the wild [18]. The leaves of half the plants were inoculated with 0.05 ml (approximately 50 cells) of *Colpoda steinii*, a medium-sized (∼50 µM) ciliate that we have used in a variety of other experiments [21],[22]. Those samples with *C. steinii* are referred to as +CS and those without as –CS. The *C. steinii* stock was originally derived from pitcher plants in the same area of the Apalachicola National Forest. The leaves were left uncovered and maintained at temperatures similar to those found in the field. Each leaf was resampled 5 days later by removing all water; the samples were immediately processed as described below. Two leaves had holes and did not contain sufficient fluid, while one sample was contaminated during processing, leaving 9 samples maintained with no protozoa and 8 with *C. steinii*.

### DNA Extraction and purification

The microbial communities in the pitcher plant fluids were concentrated by centrifugation. DNA was extracted from the pellet by suspension in a modified CTAB extraction buffer ((10% CTAB (hexadecyltrimethylammonium bromide), 1M NaCl and 0.5M phosphate buffer, pH 8) with 0.1M ammonium aluminum sulfate, 25∶24∶1 phenol∶chloroform∶isoamyl alcohol) and subjecting it to bead beating using a FastPrep-24 (MP Biomedicals, Solon, OH) at 5.5 meters per second for 45 seconds. DNA was purified using the QIAGEN AllPrep DNA/RNA Kit (QIAGEN, Germantown, MD) following the manufacturer's protocol.

### 16S rRNA Gene Sequencing and Analysis

16S rRNA genes were amplified from the purified DNA extracts in duplicate using archaeal and bacterial primers 515F and 806R, which targets the V4 region of *E. coli* in accordance with the protocol described by Caporaso et al. [23],[24] and used by the Earth Microbiome Project (http://www.earthmicrobiome.org/emp-standard-protocols/16s/), with a slight modification: specifically, the annealing temperature was modified to 60°C. Multiplexed sample amplicons were sequenced with Ilumina's MiSeq in accordance with Caporaso et al. [24] Sequences were analyzed using the QIIME version 1.7.0 [25] pipeline. Raw sequences were demultiplexed and then quality filtered using the default parameters in QIIME. Sequences are in NCBI's sequence read archive under accession number SRP048740. Sequences were then clustered into operational taxonomic units (OTUs), which was defined as ≥97% 16S rRNA gene sequence similarity, using UCLUST [26] with the open reference clustering protocol (http://qiime.org/tutorials/open_reference_illumina_processing.html). The resulting representative sequences set were aligned using PyNAST [27] and given a taxonomic classification using RDP [28], retrained with the Greengenes version 13.5 [29]. The resulting OTU table was filtered to keep only OTUs that had at least 10 sequences, and then converted to relative abundance. The differences in relativized OTU abundances in communities with and without the *C. steinii* predator were analyzed using nonparametric statistics (Mann-Whitney) to test for statistically significant differences using METAGENassist [30] and the application of a False Discovery Rate (FDR) to account for multiple comparisons. A heatmap of statistically significantly different species with and without the protozoan was generated in R with the Vegan package. Alpha diversity metrics were determined using QIIME. Specifically, Chao1, Shannon index, observed species, dominance (defined as the sum of the squares of the frequencies of each species) were determined. Statistical differences in these metrics were assessed by Student's t-test. The QIIME generated, rarefied, OTU abundances in the 17 different samples were then analyzed using non-metric multidimensional (NMDS) scaling in R using metaMDS in Vegan package, with three axes specified. P-values were derived from 999 permutations of the data.

## Results and Discussion

### Protozoa growth in *S. purpurea* leaves in the greenhouse

*C. steinii* populations were established in all the leaves where it was introduced; mean densities after 5 days were 454.4 cells/mL (standard deviation = 288.6). No *C. steinii* were found in any of the control pitchers. Although not observed in the microbial broth prior to inoculation, a protozoan contaminant, the small flagellate *Bodo menges*, was found in all of the pitchers at a density of 377.2 cells/mL (standard deviation = 229.1) at the end of our five-day study. This species is common in pitcher plants [18] and the contamination probably occurred through insufficient filtering of the field-collected samples. *Bodo menges* is known to be a competitive subordinate to *C. steinii* [22], however we found no significant difference in *B. menges* abundance between leaves with and without *C. steinii* (t = 0.47, P = 0.64).

### Community structure in *S. purpurea* inquiline Bacteria

**Figure 1:**
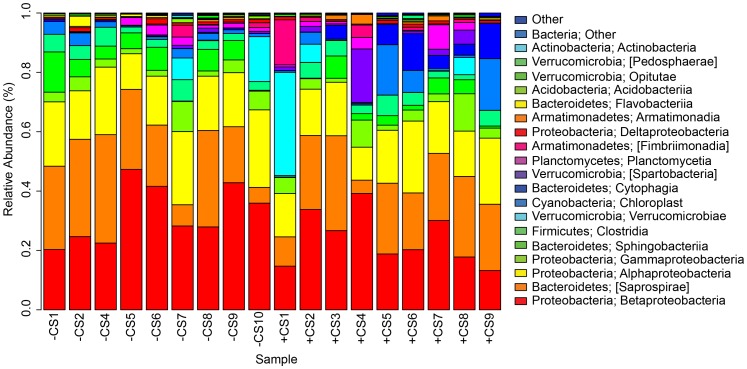
Bar graph of normalized 16S rRNA iTag sequence data. The most abundant classes are shown. Less abundant classes are summed under “Other.” Samples with *C. steinii* are referred to as +CS and those without as –CS.

Analyses of the 16S rRNA gene iTag data revealed that the microbial community in *S. purpurea* leaves, regardless of the presence or absence of a predator, was dominated by the Proteobacteria phylum, and in particular, by the sub-phyla Betaproteobacteria, Alphaproteobacteria and Gammaproteobacteria (Fig. 1). Of these three sub-phyla, Betaproteobacteria were the most prevalent (Fig. 1). Proteobacteria have been reported as the most abundant phylum in several studies of the pitcher plant microbial community, including *S. purpurea* [16],[19],[20], *S. minor* [20],[31], and in *S. alata* [20],[32]. The second most abundant phylum was Bacteroidetes (Fig. 1); both Gray et al. [19] and Krieger & Kourtev [20] also reported finding an abundance of Bacteroidetes in *S. purpurea*.

**Figure 2:**
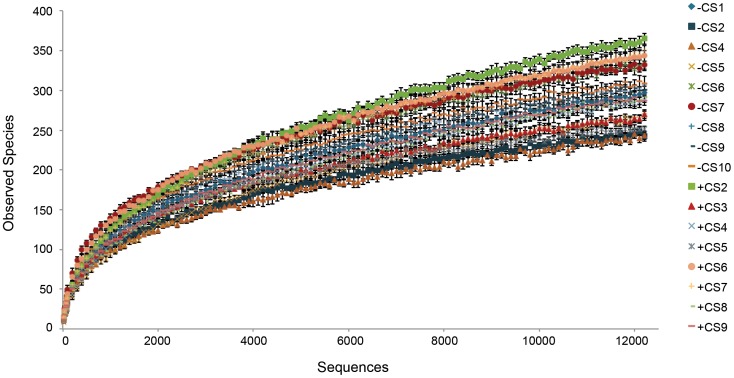
Rarefaction curve of the number of observed OTUs from 16S rRNA iTag sequence data.

Our iTag sequence data revealed 186 different genera, compared to a maximum of 19 genera using cloning and sequencing [19] in *S. purpurea* and 29 genera using a tag sequencing approach (454 pyrotag in a different host, *S. alata*; [32]). At a finer scale of taxonomic resolution, 1043 different OTUs were identified in our samples. However, rarefaction analysis (Fig. 2) revealed that even with the sequencing depth achieved here, no sample was nearing an asymptote, suggesting that greater sequencing coverage is required to capture the total breadth of microbial diversity in *S. purpurea* pitchers. Koopman et al. [32] similarly reported that they did not achieve an asymptote in their pyrotag data in *S. alata*.

### Variation in microbial community structure with and without *C. steinii*

The rarefied OTU data were further analyzed to quantify differences in the microbial communities with and without *C. steinii* (Fig. 2). No statistically significant differences in OTU richness (observed species; Table 1) with and without this predator were found. Alpha diversity was further explored to compare the microbial community in treatments with and without *C. steinii*. Chao1 [33] and Shannon diversity indices (Table 1) were not statistically different in leaves with and without the predator. Additionally, dominance values were determined for both sample types and found to be very low, nearly identical and not statistically significant for both sample types (0.08 with and without the *C. steinii* predator; dominance is on a scale of 0–1; Table 1).

**Table 1:** Alpha diversity statistics of microbial 16S rRNA gene sequence data in *S. purpurea* samples, with (+CS) and without *C. steinii* (−CS).

Sample	Chao1 (avg)	Chao1 lower bound (avg)	Chao1 upper bound (avg)	Singles (avg)	Doubles (avg)	Reciprocal Simpson (avg)	Shannon (avg)	Dominance (avg)	Observed species (avg)
**−CS1**	255.42	276.54	426.32	78.88	26.74	0.94	5.12	0.06	209.91
**−CS2**	210.74	225.91	353.27	62.27	22.17	0.93	4.98	0.07	178.82
**−CS4**	205.10	223.80	360.34	62.87	21.00	0.90	4.61	0.10	173.05
**−CS5**	229.25	245.74	383.67	74.43	26.97	0.84	3.98	0.16	186.96
**−CS6**	280.21	315.79	476.81	89.15	30.06	0.92	4.91	0.08	236.46
**−CS7**	283.51	308.78	450.68	83.54	30.49	0.97	5.94	0.03	241.86
**−CS8**	255.74	283.22	430.28	79.49	27.89	0.90	4.86	0.10	217.25
**−CS9**	256.89	280.38	418.09	78.30	28.94	0.95	5.22	0.05	218.93
**−CS10**	261.41	282.10	411.69	77.85	30.29	0.93	5.03	0.07	224.16
**+CS2**	316.20	344.82	514.68	105.04	36.81	0.94	5.19	0.06	251.06
**+CS3**	224.35	241.14	361.63	66.04	25.47	0.90	4.74	0.10	194.14
**+CS4**	242.15	263.59	401.45	78.42	28.86	0.86	4.25	0.14	202.08
**+CS5**	212.25	226.93	332.42	57.42	23.59	0.92	4.93	0.08	189.95
**+CS6**	293.32	322.86	478.70	90.50	31.32	0.96	5.63	0.04	245.16
**+CS7**	300.04	329.26	499.06	98.46	33.18	0.94	5.21	0.06	239.94
**+CS8**	249.81	274.01	432.00	81.94	27.04	0.91	4.85	0.09	201.95
**+CS9**	248.81	268.72	410.15	78.63	28.23	0.92	4.87	0.08	205.65

**Figure 3:**
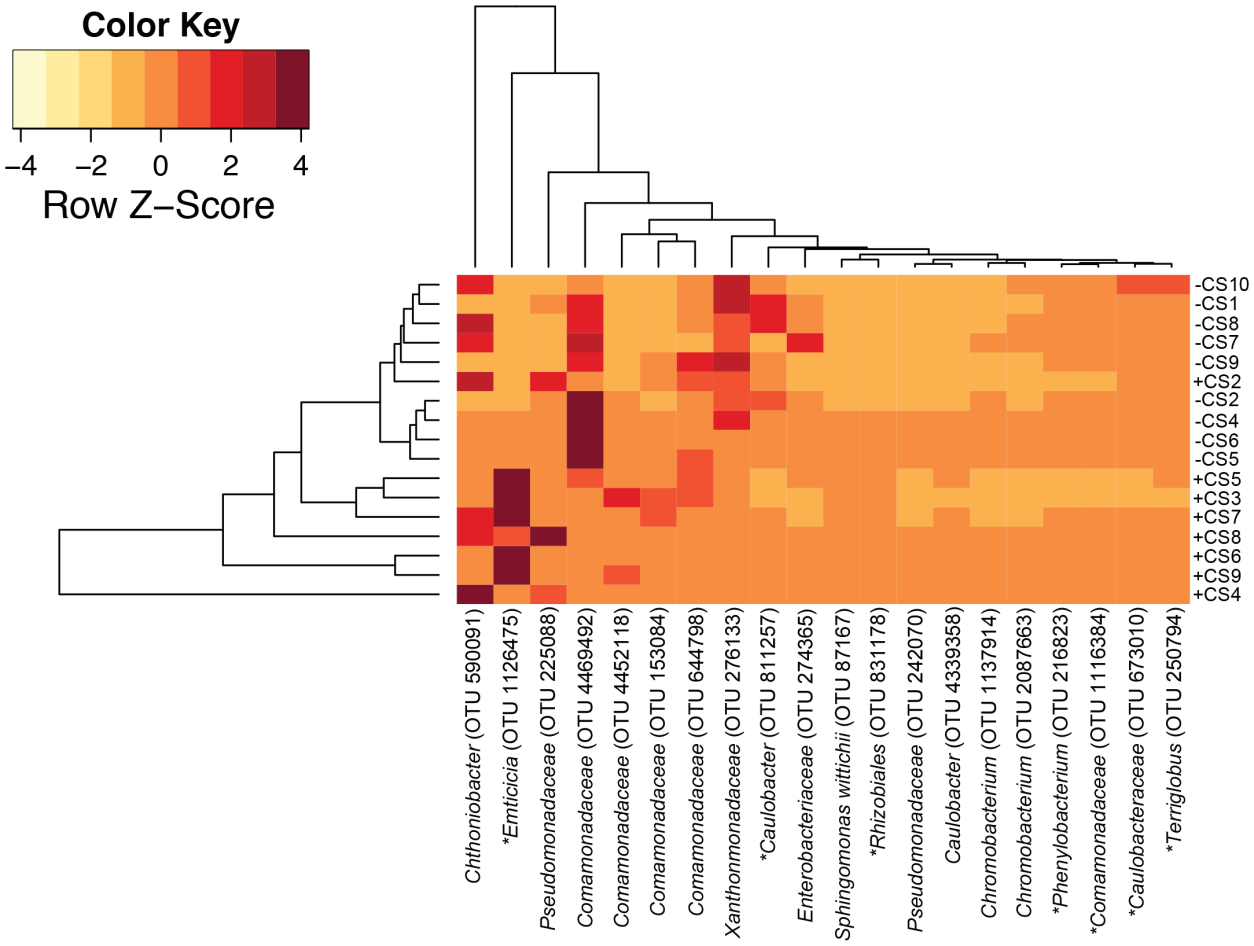
Heatmap of OTUs that were statistically significantly more abundant in samples with or without the protozoan predator (only the top ten OTUs that changed the most with protozoa and without protozoa are shown). Those OTUs that were statistically significantly correlated with an NMDS axis are indicated by an *.

To determine which of the 1043 OTUs were significantly different in samples with and without the *C. steinii* predator, we conducted non-parametric Wilcoxon tests, corrected for a FDR, on each OTU after sample normalization (relative abundance). This analysis revealed that 42 OTUs were statistically different between communities with and without the *C. steinii* predator, with those changing the most shown in Figure 3. These 42 OTUs are from a broad variety of taxonomic groups, including Proteobacteria, Verrucomicrobia, Bacteroidetes, and Acidobacteria.

Very few previous studies have quantified the effects of predators on bacterial communities (however, see [6],[12]). We found no statistical difference in species richness, dominance or overall diversity between communities with and without the protozoan predator, *C. steinii*. Previous work in *S. purpurea* revealed that protozoa may decrease bacterial diversity, but the experiments have only indirectly manipulated bacterivores through their predator, larvae of the mosquito *Wyeomyia smithii* [16],[34].

**Figure 4:**
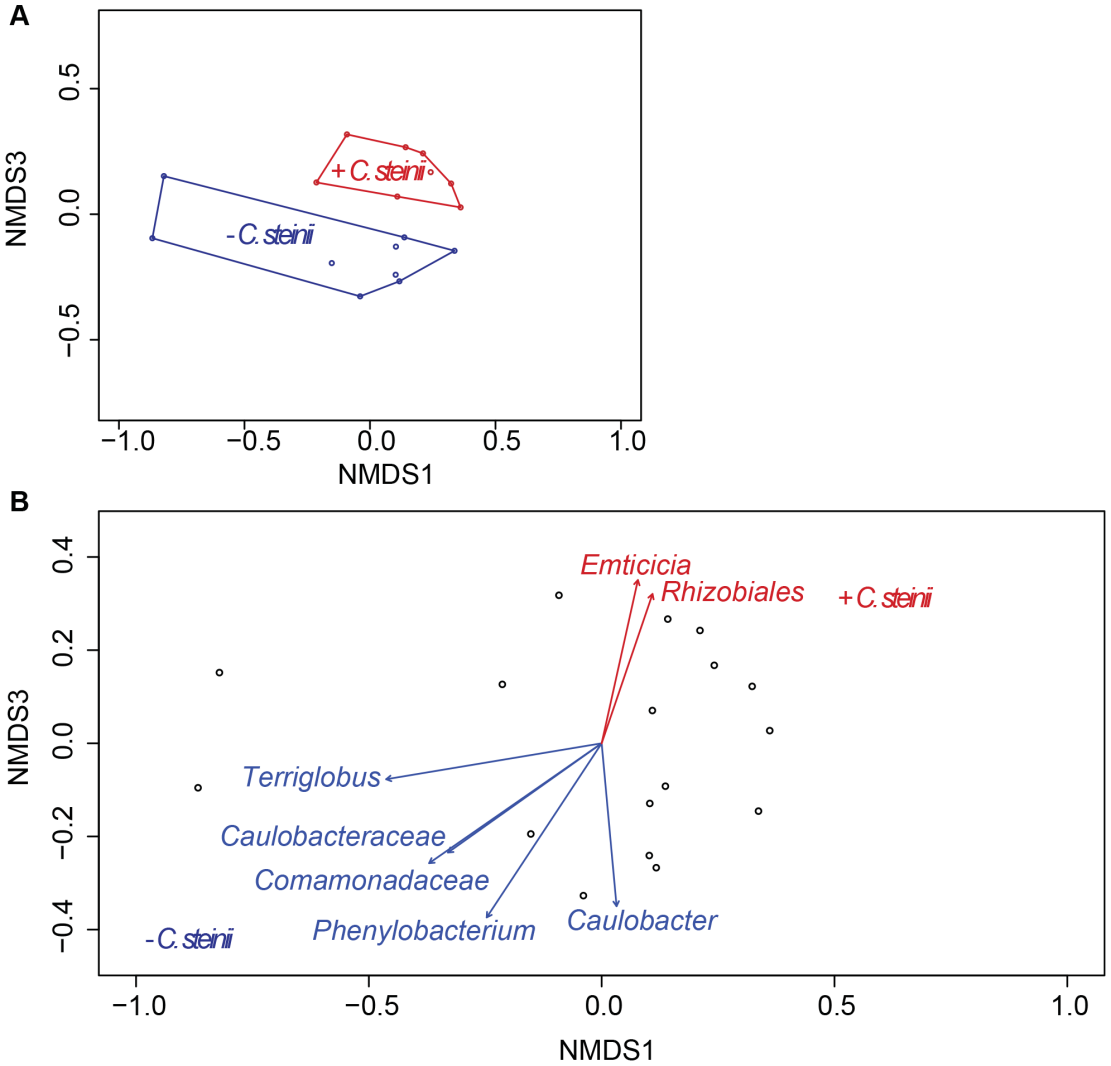
Non-metric multidimensional scaling (NMDS) ordination of normalized 16S rRNA iTag sequence data. (A) NMDS ordination of the first and third axes showing sample grouping based on the presence or absence of the protozoan predator. (B) NMDS ordination with those OTUs that were statistically significantly correlated with an axis and had a p-value < 0.03 shown by vectors.

While alpha diversity metrics were not significantly different with and without the *C. steinii* predator, beta diversity analysis revealed differences in the microbial community composition (Fig. 4), with a significant difference in scores for the third NMDS axis (ANOVA, P<0.001). Because the first two axes were not affected by the presence of the protozoan predator *C. steinii*, predation may not be the major factor determining among-leaf variation in microbial community structure, although predation clearly had a significant effect (Fig. 4a). NMDS analysis revealed that of the 42 OTUs discussed above, seven had statistically significant correlations with an NMDS axis 1 or 3 (r-value cutoff ≤0.03) (Fig. 4b). These seven OTUs are discussed in greater detail below.

### Bacterial species influenced by the presence of *C. steinii*

Seven OTUs were significantly more abundant when *C. steinii* was absent. Many of these OTUs were highly similar to species found in aquatic environments, or were closely related to plankton, both of which may indicate a planktonic lifestyle of the species in question. A planktonic lifestyle may explain why the relative species abundance was higher when no *C. steinii* was present - because it is unable to seek refuge by attaching to a surface, such as a soil particle, to escape protozoan predation. A more detailed discussion regarding this hypothesis follows below. We acknowledge, however, investigating this hypothesis requires additional experiments beyond those described herein. Further, we recognize that close phylogenetic relationships (16S rRNA genes) between uncultured and cultured species is not indicative that the species in question is endowed with a particular attribute.

A *Comamonadaceae* species (OTU 1116384), a member of the Betaproteobacteria, was more abundant in leaves without predators than those with predators (Figs. 3 & 4b). This *Comamonadaceae* species was 99% similar to sequences retrieved from a wide variety of habitats, ranging from marine (Acc. KC872921) and freshwater (Acc. KF827131), to acidic water from a copper mine (Acc. KF287732) and to intestinal microbiota in migrating shorebirds in the Delaware Bay [35]. *Roseateles depolymerans*, a motile, rod shaped, bacteriochlorophyll containing microorganism isolated from freshwater [36], was closely related (99%) to the *Comamonadaceae* species. Given that the abundance of the *Comamonadaceae* species discussed here was statistically significantly higher without the predator, suggested it may be subject to protozoa grazing in the pitcher fluids. Its high similarity to species found in aquatic environments may indicate a planktonic lifestyle, which, as discussed above may preclude predator avoidance by particle attachment.

The Alphaproteobacteria, *Caulobacteraceae* species (OTU 673010) was more abundant in leaves without *C. steinii* than in those with it (Figs. 3 & 4b). This species was highly similar to microorganisms from several disparate environments. For example, it was 100% similar to microorganisms associated with insects (Acc. JQ894899 and [37]), forest soil [38], marine (Acc. KC001312) and freshwater [39] environments. *Caulobacteraceae* (OTU 673010) was 97% similar to several cultured representatives including *Phenylobacterium lituiforme*. *P. lituiforme* is a motile, facultative anaerobe isolated from a subsurface aquifer [40], thus may have a planktonic lifestyle. The close relationship of the *Caulobacteraceae* (OTU 673010) species with *P. lituiforme* and to microorganisms from other aquatic environments may indicate that it has a planktonic lifestyle, which could make it more susceptible to predation.

Similarly, the Alphaproteobacteria, *Caulobacter* (OTU 811257) was more abundant when no *Colpoda steinii* was present (Figs. 3 & 4b). This species was 100% similar to those from a wide variety of habitats, including coral (Acc. KJ601398), seawater (Acc. KF786782) and root nodules (Acc. KF596691). It was 100% similar to the cultured representative, *Caulobacter crescentus*, which is a motile species typically found in aquatic environments with low nutrient concentrations [41] - an environment found within the *S. purpurea* pitcher.

*Terriglobus* (OTU 250794) a species in the phylum Acidobacteria, was more abundant in leaves without *Colpoda steinii* (Figs. 3 & 4b). Sequences that were 100% similar to *Terriglobus* (OTU 250794) included microorganisms from sugarcane stem (Acc. KF241163), Nepenthes pitcher plants (Acc. JX532061), *Eisenia fetida* egg capsules [42] and acidic Sphagnum peat [43]. *Terriglobus saanensis* strain SP1PR4 [44] was highly similar (98%) to the *Terriglobus* (OTU 250794). *Terriglobus saanensis*, isolated from Artic tundra soil, is a non-motile, rod-shaped, aerobic bacterium [44]. The fact that our *Terriglobus* (OTU 250794) species is possibly soil associated, is not in agreement with the hypothesis we proposed above. The high sequence similarity of this species with Bacteria from other pitcher plants, suggested that it is a ubiquitous member of pitcher plants, and warrants more direct attention in future research, particularly in regards to its lifestyle - planktonic or particle associated.

Finally, the Alphaproteobacteria, *Phenylobacterium* (OTU 216823) had a higher relative abundance in leaves without *C. steinii* than those with it (Figs. 3 & 4b). This species was 100% similar to microorganisms associated with plant roots (e.g. Acc. FM956526) and a wetland [45]. Further, it was 98% similar to *Phenylobacterium falsum*, which was isolated from alkaline groundwater [46]. *P. falsum* is a small rod-shaped, aerobic, heterotrophic non-motile bacterium [46]. The close relationship of *Phenylobacterium* (OTU 216823) species to *P. falsum* may indicate that it is planktonic, but non-motile, which could render it difficult to escape predation-even more so than the potentially planktonic, motile species discussed above.

### Bacterial species that may be capable of escaping *C. steinii* predation

The Alphaproteobacteria, *Rhizobiales* (OTU 831178), was more abundant in the leaves with *C. steinii* than in those without (Figs. 3 & 4b). This species was 100% similar to microorganisms found in a wide variety of environments including, solanaceous crops [47], alluvial aquifers of the Makyeong River [48] and bacterial communities associated with spores of *Gigaspora margarita* (Acc. EU589423). The cultured species *Bosea thiooxidans* was 99% similar to the *Rhizobiales* species (OTU 831178) [49]. *B. thiooxidans* is a rod-shaped, motile bacterium isolated from soil [50]. This contrasts with the species discussed previously, in that the closest cultured representative is soil-associated, not planktonic. Our hypothesis that the protozoa predator may target planktonic Bacteria, rather than particle, or soil-associated species is supported. Alternatively its abundance may have been shaped by an interaction with other Bacteria that decreased in abundance when a predator was present. However, these hypotheses were not directly tested here, and require additional future analyses.

*Emticicia* (OTU 1126475), a *Cytophagia* in the Bacteroidetes phylum, was more abundant in samples with *C. steinii* than those without (Figs. 3 & 4b). The most similar (100%) sequences to this *Emticicia* species were retrieved from pasture soil (Acc. JN976780) and from the isolate *Emticicia ginsengisoli* strain Gsoil 085 [51]. The aerobic, non-motile, rod-shaped bacterium, *E. ginsengisoli* strain Gsoil 085, was isolated from soil samples in a ginseng field [51]. As discussed previously, Bacteroidetes is a major part of the microbial community of *S. purpurea* pitcher fluid [19],[20]. Gray et al. [19] reported that Bacteroidetes was a significant member of the microbial community in *S. purpurea* fluids, including those pitchers sampled at the Apalachicola National Forest, from where we obtained pitcher fluids for our greenhouse experiments. These authors suggested, however, that the variability in the abundance of Bacteroidetes from leaf-to-leaf may not result from the grazing pressure, but rather other environmental factors [19]. Our results suggest that predation does influence the abundance of a particular Bacteroidetes species. This finding further supports our hypothesis, in that the *Emticicia* (OTU 1126475) species, closely related to the soil isolate *E. ginsengisoli*, may not have a planktonic lifestyle, and may be able to attach to surfaces such as soil particles, thereby avoiding predation. Further, Gray et al. [19] highlighted the role Bacteroidetes play in degrading organic matter and in producing extracellular enzymes, which may be important mechanisms by which nutrients are made available to *S. purpurea*. Thus the ability of a Bacteroidetes to avoid predation may directly influence the fitness of *S. purpurea*.

## Conclusions

The goal of this study was to determine the predator-prey effects of the protozoan, *C. steinii*, on the microbial community in *S. purpurea* pitcher fluids. Our results revealed that, while grazing by this protozoan in *S. purpurea* pitcher fluids resulted in statistically significant shifts in the relative abundance of 42 bacterial species, there was no change in overall microbial community richness or diversity. Of these 42 species, those that had a statistically higher relative abundance without *C. steinii* present were generally closely related to motile, planktonic species, leading us to hypothesize that, in our experiments, protozoa targeted microorganisms residing in the pitcher fluids, rather than those associated with particles, such as soil. This hypothesis is in agreement with our findings: that those microbial species that were more abundant in the samples with *C. steinii* described herein, were closely related to particle associated cultured representatives. Our findings help to resolve, at a microscale level, the effects perturbations have on trophic structures by the addition or removal of the protozoan predator. Although, it is likely that there are many factors that influence prey selection and predator avoidance, testing our hypothesis in future experiments is feasible. For example, differentiating between the microbial communities associated with particles and those associated with the pitcher fluids may reveal that protozoa do indeed target the plankton.
